# He was only thirty one

**DOI:** 10.1017/S1478951526102582

**Published:** 2026-04-30

**Authors:** Nidha Shapoo, Noella Boma

**Affiliations:** Department of Medicine, New York Health + Hospitals/Metropolitan, New York Medical College, New York, NY, USA

I remember the moment I opened his chart for the first time in Epic.

I was covering the team over the weekend. Among the many admissions in that unit: acute heart failure, asthma exacerbation, alcohol withdrawal, his diagnosis made me pause:

Carcinoma of unknown primary with bone metastases, spinal cord compression, and paraplegia.

He was only thirty-one.

Younger than me. That fact unsettled me more than I expected.

I spent more time reviewing his chart, trying to understand how a young man could suddenly develop a disease that most people face decades later. His symptoms started only 3 weeks earlier: back pain that worsened quickly, weakness in his legs, and then the sudden loss of movement.

He had recently moved to the United States from Santo Domingo with a handful of dreams, like many of us who leave our countries believing the future will open more generously elsewhere. Instead, he lay in a hospital bed with legs that no longer moved.

When I entered the room, the weight of those words met a young man who still looked surprisingly healthy on his face.

He had serene eyes. He spoke in a gentle, courteous tone.

But his legs lay still beneath the hospital blanket, and a urinary catheter protruded from beneath the sheets. He had lost control of his bowels and bladder.

Just weeks earlier, he had been walking.

I introduced myself and explained that I would be caring for him during the day. I asked about his symptoms and worked to keep him comfortable. I adjusted his opioids, anti-emetics, and laxatives. I confirmed that physical therapy had been consulted.

Then I moved on to the other patients waiting to be seen.

The long call finally wrapped up. Sign-outs were completed, and the hospital returned to its usual routine of admissions and discharges.

But his face stayed with me.

Among all the patients I had seen that weekend, he became the one whose progress I wanted to know.

I occasionally checked his chart during quiet moments between patients, even as I rotated through different specialties.

A quick check between tasks with a curiosity I could not fully explain.

At the time, I told myself I was following his clinical course. But in retrospect, I believe I was trying to understand something I didn’t yet have words for.

His hospital course was challenging.

High calcium levels, fever, nausea and vomiting, and pleural effusions. Chemotherapy was started for palliation, in the hope that the cancer, still without an identifiable primary, might respond.

Days turned into weeks. Weeks turned into months.

He spent over 2 months in the hospital.

It was because of him that I chose malignant spinal cord compression for my morning report presentation. I wanted the residents to learn how to recognize and respond to such cases if they ever encount them at the bedside.

I started my hematology and oncology rotation shortly after.

He was still admitted.

This time, I was no longer observing from afar. I became involved in his care, writing daily progress notes and examining him during rounds.

I observed the swift decline of his illness. The cancer was unforgiving.

Chemotherapy failed. His body began to change rapidly. The young man I initially met, whose face still radiated health despite his paralysis, grew noticeably weaker in less than 2 months.

The discussions eventually changed.

Treatment was no longer effective. The disease continued to worsen. The focus shifted to family meetings and discussions about end-of-life care.

These conversations are challenging for the patient who must hear them, for the family who must accept them, and for the physician who must find the right words to discuss a future that will be shorter than anyone had hoped.

The choice to move to hospice care at home was made after thorough discussions with his family.

Throughout everything, he stayed remarkably composed. He seldom complained.

Whenever we asked how he felt, he almost always gave the same answer.

“I’m fine.”

My rotation in hematology and oncology had ended by then, and I hoped he would return home safely.

I went back to working night shifts.

My pager rang during the first night shift while I was still doing the handover.

“Doctor, the patient is breathing rapidly,” the nurse said over the phone. Oxygen saturation is dropping. Blood pressure is 60 over 40.

I hurried to the ward without reviewing the patient’s information.

When I stepped inside the room, I froze. It was him.

I never thought I would see him again, especially now in his final hours.

The room was packed. Nearly 10 family members gathered around the bed: his parents, sisters, and friends. The air was heavy with the quiet panic that fills a room when everyone senses what is about to happen.

He acknowledged me and smiled.

His mind was clear, but his body was extremely wasted, reduced to skin and bones. As I moved closer, his gaze followed me.

“How are you feeling?” I asked softly as I leaned in. Are you in pain?

He shook his head. “No”

His sister approached me, her voice trembling.

Doctor, we can’t see him like this, she said. Is there any way you can speed this up so he can pass peacefully?

The words felt heavy.

It was the first time I was asked a question like this.

I paused there, unsure for a moment, not because I didn’t know the answer, but because I had never felt the question so deeply. In a profession where we are taught to save lives, I was being asked if there was a way to hasten the end of one.

I gently explained that while we couldn’t speed up death, we would do everything possible to keep him comfortable, help his breathing, relieve pain, and make sure he was not in suffering.

After leaving the room, I went back to the work desk to review labs, answer pages, and admit new patients.

An hour later, the phone rang.

“Doctor,” the nurse said softly, “the patient expired.”

In that quietness, what stayed with me was not just the loss but also the privilege of witnessing his journey.

As physicians, we often see patients only in fragments, but with him, I experienced almost the entire journey.

I saw a face that radiated health and dreams just beginning. I saw the shock of the diagnosis. I witnessed the relentless advance of his cancer, with complications unfolding one after another, his body weakening day by day, his strength quietly slipping away right before my eyes. I watched him deteriorate as the disease took more of him than medicine could restore. Yet through it all, he remained surprisingly calm. There was no anger, no frustration, no bitterness, only a quiet acceptance of the fragility that had suddenly entered his life.

Even in those final moments, when his fragile body fought for air, and the room was filled with the panic of those who loved him, his eyes stayed steady and aware as if he had already come to terms with something the rest of us were still trying to understand.

From the day he arrived until his last breath, he faced his illness with a calmness I rarely see, even in those who have lived much longer. He didn’t let his illness take away who he was, even if his body had failed him.

I had always believed that my role was to treat, to fix, to move things forward. But with him, there came a point where there was nothing left to fix.

He was only thirty-one.

And without ever intending to, he taught me something I wish we emphasized more in training: that medicine isn’t always about changing the outcome, but about how we show up when the outcome can’t be changed.

